# Patients with Chronic Spinal Cord Injury Exhibit Reduced Autonomic Modulation during an Emotion Recognition Task

**DOI:** 10.3389/fnhum.2017.00059

**Published:** 2017-02-08

**Authors:** Gonzalo Varas-Díaz, Enzo P. Brunetti, Gonzalo Rivera-Lillo, Pedro E. Maldonado

**Affiliations:** ^1^Instituto de Ciencias Biomédicas, Facultad de Medicina, Universidad de ChileSantiago, Chile; ^2^Centro de Estudios Integrados en Neurorehabilitación, Clinica Los CoihuesSantiago, Chile; ^3^Departamento de Kinesiología, Facultad de Medicina, Universidad de ChileSantiago, Chile; ^4^Biomedical Neuroscience Institute (BNI), Universidad de ChileSantiago, Chile

**Keywords:** autonomic nervous system, social cognition, heart rate variability, spinal cord injury, emotional processing

## Abstract

Spinal cord injury (SCI) is a devastating event for individuals, who frequently develop motor and sensory impairment as well as autonomic dysfunction. Previous studies reported that autonomic activity plays a major role in social cognition and that difficulties in the ability to interpret social information are commonly observed in a variety of mental disorders, which in turn correlate with a poor autonomic nervous system (ANS) regulation. It is well established that subjects with SCI have an alteration in ANS regulation mechanisms. We hypothesized that subjects diagnosed with SCI, who are experiencing a period of adaptation and socio-labor insertion suffer alterations in an emotion recognition task, a component of social cognition, which correlate with poor ANS regulation. We evaluated ANS function by measuring the heart rate variability (HRV) in 18 healthy subjects and 10 subjects with SCI. A 5-min baseline HRV was compared to a task period while performing The reading the mind in the eyes test (RMET). We found that while both groups have similar general performance in the test, healthy subjects responded with greater certainty during the RMET. This level of certainty during the RMET was positively correlated with baseline HRV measures in this group. Also, the group of healthy subjects exhibited higher HRV at baseline than participants with SCI. Finally, the changes in HRV between baseline and task condition were significantly higher in healthy individuals than in SCI participants. Our results show that patients with SCI have low levels of autonomic regulation mechanisms which may promote social cognition problems during their reinsertion to daily life.

## Introduction

Spinal cord injury (SCI) is a devastating event that results in disturbances to normal sensory, motor, or autonomic function. This condition ultimately impacts a patient’s physical, psychological and social well-being (Rossignol et al., 2007). SCI annual incidence and prevalence is high worldwide. In the United States, the prevalence is the highest, estimated in 906 per million, while the incidence in other countries varies between 49 per million in New Zealand, and 8 per million in Spain (Singh et al., 2014). In most countries, the incidence of traumatic SCI is higher in people younger than 30 years old (Furlan et al., 2013), with a lifespan of over 15 years, which is associated with high personal and social costs (Priebe et al., 2007).

In recent years, some results of studies in people with SCI have impacted significantly on their health, physical capacity and quality of life. The pathophysiology of SCI in acute stages (Dietz, 2010), the impact of different types of therapies with stem cell transplantation (Valenzuela et al., 2012), and the role of physical therapy in rehabilitation of people with SCI (Angeli et al., 2014) have been the subject topics of great interest in the last years. While these lines of research have contributed significantly to optimize the processes of rehabilitation related with impairment and activity limitations of people with SCI, other aspects of research related to social cognition and social participation have been less investigated. One of these issues relates to how changes in bodily responses and peripherical arousal, primarily determinate by autonomic activity, may affect emotional processing, which in turn, may have negative implications in SCI subjects social insertion processes (Inoue et al., 1995; Appelhans and Luecken, 2006). In this context, it has been reported that mood disorders in people with SCI are associated, in turn, with a variety of poorer outcomes including increased hospitalization periods, and secondary medical conditions, as well as decreased social integration quality of life, and self-care dependency (Munce et al., 2015).

The perception and generation of responses to intentions and behaviors of others are known as social cognition (Green et al., 2008). The recognition of facial expressions and the ability to infer the likely mental states of other people is known as Theory of Mind (TOM), an important topic of social cognition, which reflects deeply functional social capacity (Baron-Cohen et al., 2000; Bora et al., 2006). The emotions that people experience while interacting with their environment are related to constant physiological changes (Damasio et al., 2000). A key system involved in the generation of this physiological arousal is the autonomic nervous system (ANS), which is responsible for balance between sympathetic and parasympathetic activity (Berntson et al., 2003; Appelhans and Luecken, 2006; Rodrigues et al., 2016). One way used to explore the relationship between the ANS activity, and emotional processing is the analysis of Heart rate variability (HRV; Appelhans and Luecken, 2006). HRV is considered an effective tool to quantify the residual cardiovascular sympathovagal regulation after SCI and was highly reproducible in this population (Malmqvist et al., 2015). Also, HRV analysis is emerging as an objective measure of regulated emotional response (appropriate timing and magnitude), and functions associated with social cognition and TOM (Pumprla et al., 2002; Quintana et al., 2012).

In the last years some studies have described that people with different types of psychiatric disorders such as autism (Van Hecke et al., 2009; Bal et al., 2010), depression (Lee et al., 2005; Kemp et al., 2010), and post-traumatic stress show alterations in the field of social cognition that correlate with a decrease in the HRV or imbalances in the autonomic regulation. A low HRV reflects an ANS that has a reduced ability to modify the influence of the sympathetic and parasympathetic system as required by a particular situation; that means a poor autonomic flexibility (Thayer and Lane, 2000).

There is evidence that sympathetic cardiovascular control in SCI above spinal segment T6 is impaired or even absent (Malmqvist et al., 2015). Consequently, cardiovascular disorders may occur such as reduced HRV, bradycardia, arterial hypotension and autonomic dysreflexia (Oh and Eun, 2015; Serra-Año et al., 2015). Also, studies have shown that although sympathetic innervation is preserved in SCI patients below T6, there may be autonomic disorders, such as decreased parasympathetic influence in HRV (Bunten et al., 1998; Claydon and Krassioukov, 2008; Jan et al., 2013). The aim of this study was to determine whether autonomic alteration of persons with SCI affects their performance in a social cognition task. We hypothesized that this type of basal activity of the ANS decreases autonomic flexibility that has been described as favorable for social cognition tasks. To test our hypothesis, HRV was measured as an autonomic marker, at rest and during the performance of the reading the mind in the eyes test (RMET). This later test assessed the affective component of the TOM, in healthy subjects and persons with SCI, who were experiencing a period of adaptation and socio-labor insertion.

## Materials and Methods

### Subjects

We recruited 18 healthy subjects and 10 subjects with SCI ASIA Impairment Scale (AIS) A and B, diagnosed with paraplegia, who were experiencing a period of adaption and socio-labor insertion (see demographics in Table 1). All subjects with SCI had ended his rehabilitation process and were working or studying (period of adaptation and socio-labor insertion). Exclusion criteria included psychiatric illness diagnosed by psychology department of Los Coihues Clinic after application of Hamilton Anxiety Rating Scale (HARS) and Hamilton Depression Rating Scale (HDRS), current use of antidepressants, the presence of autonomic dysreflexia in the last 6 months, and any other serious medical condition (e.g., cardiovascular disease). To avoid any confounding influences of other substances on psycho physiological functioning, participants were asked to abstain from consumption of caffeine, cigarettes and alcohol on the day of testing. Basic demographic data of the patients are listed in Table 1. The healthy control group was statistically similar in age and gender to the patient group. This study was carried out in agreement with the recommendations of the Comité de Ética de Investigación en Seres Humanos from the Facultad de Medicina, Universidad de Chile, with written informed consent from all subjects. All subjects gave written informed consent following the Declaration of Helsinki. The protocol and consent form was approved by the Comité de Ética de Investigación en Seres Humanos.

**Table 1:** **Patients’ demographic data**.

Subject	Age	Gender	Neurological level	Months after lesion	ASIA
1	36	M	T4	19	A
2	24	M	T4	46	A
3	24	F	T10	18	B
4	23	F	T10	21	A
5	24	F	T6	14	B
6	44	M	L2	32	B
7	30	M	T4	16	A
8	28	M	T6	13	A
9	31	M	C8	18	B
10	39	F	T10	51	A

### Cognitive Task and Heart Rate Measurements

**Figure 1:**
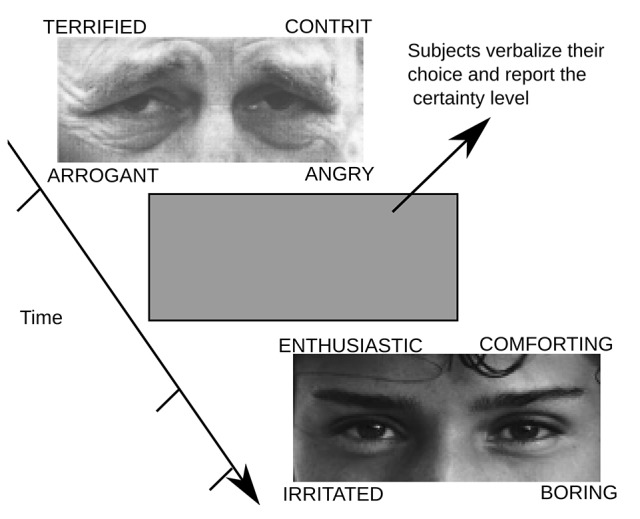
**An example of the experimental sequence.** Participants must respond the reading the mind in the eyes test (RMET). They must choose the answer that in their opinion best represents the emotional state or what the person in the photograph is thinking. Answers are shown in the test image. Each image could be seen by participants for an unlimited time; once they chose a response they were asked to press a button in a joystick that gave way to a gray image. When the screen turned gray, participants must verbalize their choice and also report the level of certainty with which they were responding (1 = unsure, 2 = fairly sure and 3 = sure).

Participants completed the RMET, which assesses the affective component of the TOM, to determine emotion recognition aptitude (Baron-Cohen et al., 2001). All measurements were recorded between 15 and 18 pm. Participants are presented with 36 images of the eye region of different faces, providing four options for each image. Participants are instructed to answer each response as quickly as possible. After the response, the image was replaced by a gray screen and participants reported the certainty level of their responses, where 1 was unsure, 2 fairly sure, 3 very sure. The images were presented on a 21-inch (Viewsonic p815), with a distance between the subjects and the monitor of 57 cm. Each image was displayed without a time limit, and when participants had an answer, they pressed a button that turns the screen gray. At this moment, participants verbalized the answer and reported the certainty level of their responses. Then, the subjects pressed a button to trigger the next image presentation. The time required for the task was around of 1 h. The temperature of the room where the experiment was performed was 20°C. An example of images and choices presented in the task are depicted in Figure 1. The correct response was computed over all trials conducted by the subjects, in all three sessions. The chance level was 25% correct.

The electrical heart activity was measured by a heart rate monitoring system (Polar RS800CX, Polar Electro Oy, Kempele, Finland) using a sampling rate of 1000 Hz, which wirelessly receives HR data from a chest strap (two-lead) worn by the participants (Weippert et al., 2010). The raw data was extracted from a text file and imported into Kubios (version 4.0, 2012, Biosignal Analysis And Medical Imaging Group, University of Kuopio, Finland, MATLAB). After a resting period of 5 min, a new 5-min quiet sitting period was acquired at the beginning of the assessment, to be used as the baseline HRV. Then, cardiac signals were acquired again during the performance of the RMET. Participants were breathing spontaneously during the recording period. The sitting position was chosen because, due to the extensive functional loss after SCI, affected individuals use a wheelchair and remain in the seated position most of the time (Serra-Año et al., 2015).

### Data Analysis

HRV was computed, as an autonomic marker, in all participants. HRV analyses were performed in the time and frequency domains. We employed Kubios to calculate high frequency (HF), and low frequency (LF) HRV power (HF, 0.15–0.4 Hz; LF, 0.04–0.15 HZ; normalized units) using the Fast Fourier transform, and the root means square of successive differences (RMSSD) of RR intervals. The frequency domain of HRV methods uses the power spectral density that measures how power distributes as a function of frequency (Malik et al., 1996). In the frequency domain, LF and HF mostly reflect sympathetic and vagal modulation of HRV, respectively (Pumprla et al., 2002; Jan et al., 2013). Also, RMSSD were regarded as the primary indexes of the cardiac vagal tone.

### Statistical Analysis

We examined whether all data obtained in this study were normally distributed, using the Shapiro-Wilk test. Then, the data were analyzed using a one-way analysis of variance (ANOVA) with repeated measures in each group. Differences were considered statistically significant at *p* < 0.05. The Spearman rank correlation test was used to analyze relationships between different HRV variables and performance in RMET. A *post hoc* power analysis was conducted utilizing the statistical software programs G*Power (version 3.1.9.2 Dusseldorf Germany).

## Results

### Behavior in the RMET Test

**Figure 2:**
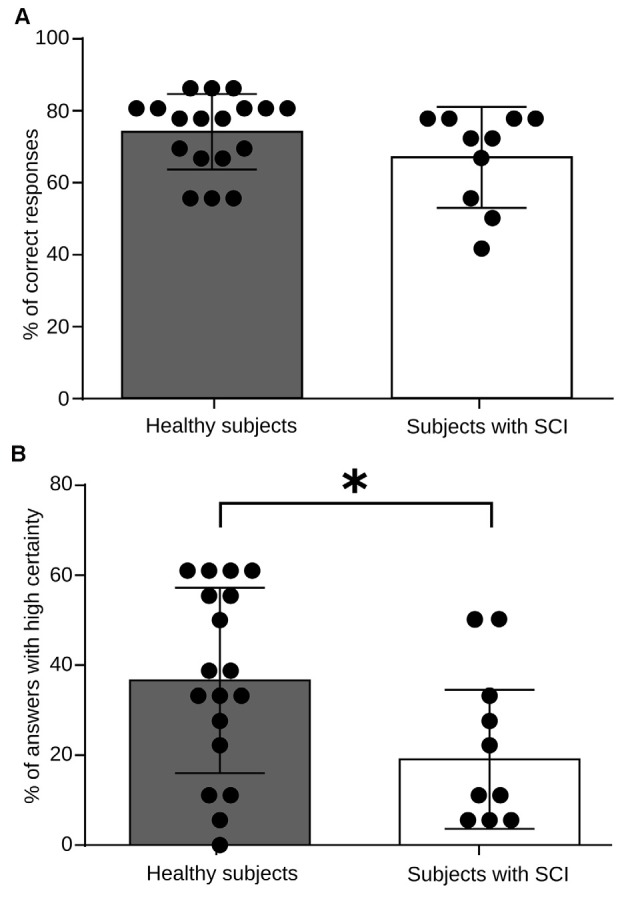
**RMET performance for healthy subjects and spinal cord injury (SCI) patients. (A)** Total performance in the task. The bars indicate the average measure for high frequency (HF) heart rate variability (HRV) power, and each black dot represents values obtained for every individual. No significant differences in performance were observed between groups (*p* = 0.0860). **(B)** Certainty level in the task responses. As above, the bars indicate the average measures and each black dot represents values obtained for every individual. Healthy subjects reported a greater number of responses with a high level of security (*p* = 0.025). *p < 0.005.

To assess the existence of differences in performance during the RMET between the group of healthy subjects and the group of persons with SCI, we computed the percentage of correct answers as well as the percentage of answers provided with a high level of certainty. Chance level in each trial was 25% (four alternatives per trail). We presented 36 images in each, session which was performed three times. Thus the correct response percentages were computed considering 108 trials as the 100%. As shown in Figure 2A, we found that both the groups, patients, and healthy control group, exhibit similar performance. We found no significant differences in the number of correct answers between the two groups during RMET (*p* = 0.086). However, when we asked their level of certainty on their responses, we found that the group of healthy subjects answered a larger percentage of trials with the highest level of certainty (*p* = 0.025; Figure 2B). These results indicate that patients can recognize the emotional content of the images used in the test as well as healthy control do, but the most of the time, they do so unsure of their responses.

### Autonomic Measurements

We found that during the RMET, the basal heart rate of both, the healthy and SCI groups did no differ from baseline (Figure 6). Therefore, to explore further the autonomic activity in healthy subjects and persons with SCI resulting from the proposed experimental design, two variables of HRV were analyzed; the LF/HF power of HRV and the RMSSD.

**Figure 3:**
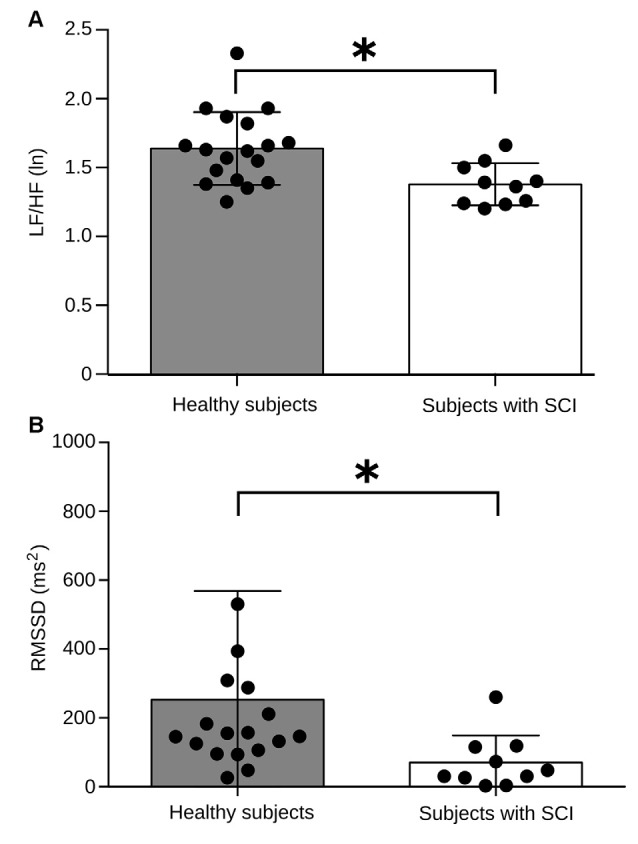
**Comparison of basal autonomic activity between healthy subjects and subjects with SCI. (A)** Low frequency (LF)/HF HRV power between both experimental groups. The bars indicate the average measure for LF/HF HRV power, and each black dot represents values obtained for every individual. Healthy subjects have an LF/HF power of HRV significantly higher compared to the group of subjects with SCI (*p* = 0.0025). **(B)** Root means square of successive differences (RMSSD) values between both experimental groups. Healthy subjects have an RMSSD significantly higher compared to the group of subjects with SCI (*p* = 0.0041). Brackets above each bar indicate SD. *p < 0.005.

Because LF/HF HRV is regarded as an important indicator of the sympathovagal tone, we initially assessed the ANS regulation mechanism during the baseline session. The results of all individual and the population average are shown in Figure 3. Healthy subjects had significant differences in this measure with an average LF/HF HRV power of 1.63 (ln), compared with 1.37 (ln) in the patients’ group (Figure 3A). A similar result was observed when we computed the RMSSD. It has been established that the RMSSD indicator, derived from statistical analysis of continuous measurement of HRV, corresponds to a parasympathetic activity index (Pumprla et al., 2002). We found that RMSSD values in the healthy group average 37.1 ms, compared to 18.5 ms in the patient group (Figure 3B). These results indicate that during baseline or resting condition, patients exhibit a reduced autonomic regulation capacity regarding the control group.

**Figure 4:**
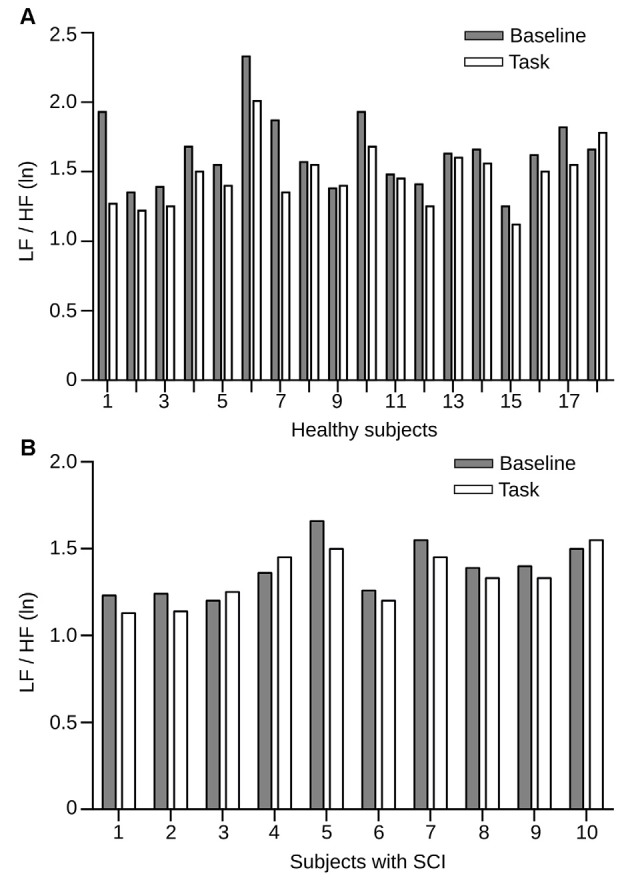
**HRV measures in healthy individuals and SCI patients before and during the RMET task. (A)** LF/HF HRV power between rest condition (gray bars) and task (white bars) for each subject from the healthy group, sorted by subject. All but three individual incremented their LF/HF HRV power during the task. Here, the differences in power were statistically significant (*p* = 0.0218). **(B)** LF/HF HRV power for the SCI group. Conventions of color are the same as in **(A)**. Here the differences in power were also statistically significant (*p* = 0.0114).

One of the objectives of this study was to test whether SCI patients differ in their autonomic regulation mechanisms while engaging in a social cognition task. Therefore, we used the LF/HF HRV measures during the RMET and compared them to the baseline period. It is well established that, in general, tasks that require attention and cognitive effort show a decrease in HRV due to increased sympathetic activity. The changes in HRV during the RMET regarding baseline condition in the two groups of study are shown in Figure 4. We found in the control group that HRV decreases significantly compared with baseline (*p* = 0.001; Figure 4A). In contrast, LF/HF HRV index in the patient group resulted in no significant changes between the baseline and task (*p* = 0.107; Figure 4B). Despite the small patient number, the *post hoc* power analyzed after data collection was 90.8%. These results demonstrate that unlike healthy individuals, SCI patients do not exhibit the normal autonomic changes expected during a social cognitive task.

### Correlation between Autonomic Measurements and Response Certainty

**Figure 5:**
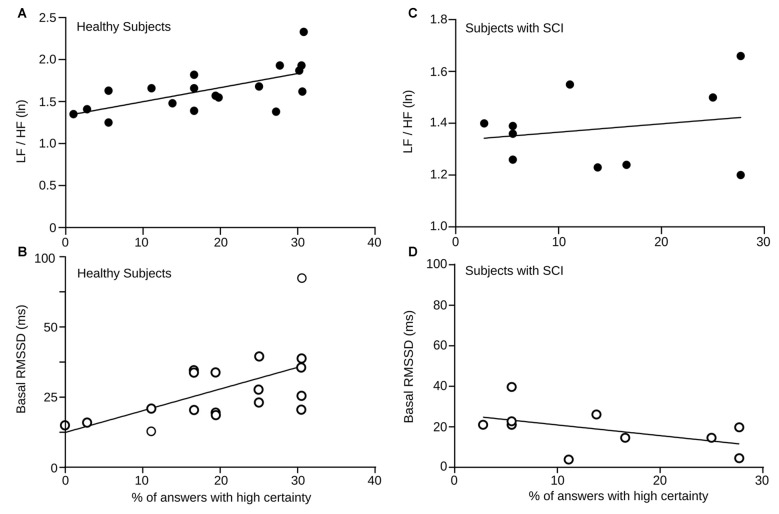
**(A)** Baseline LF/HF HRV power vs. percentage of answers given with high certainty (= 3) by every healthy participant depicted here by a black dot. The solid line corresponds the computed linear correlation. *r* = 0.5, *p* = 0.032. **(B)** Baseline RMSSD values vs. percentage of answers given with high certainty (= 3) by each healthy participant. The solid line corresponds the computed linear correlation. *r* = 0.5, *p* = 0.013. **(C)** Baseline LF/HF HRV power vs. percentage of answers given with high certainty (= 3) by each SCI subject. The solid line corresponds the computed linear correlation. *r* = 0.39, *p* = 0.26. **(D)** Baseline RMSSD values vs. percentage of answers given with high certainty (= 3) by each SCI subject. The solid line corresponds the computed linear correlation. *r* = 0.48 *p* = 0.1477.

We found differences in autonomic activity during the execution of RMET between the group of healthy subjects and the group of persons with SCI. To further explore the relation between this cognitive task and the autonomic activity, we compared the baseline LF/HF HRV and RMSSD values of healthy controls and SCI patients to their respective certainty level reported during the RMET task. Figure 5 shows correlation measures of basal LF/HF HRV and RMSSD values with the percentage of total answers delivered with high certainty levels in healthy and SCI subjects. For healthy subjects, we found a positive correlation between both measure with the answer certainty (LF/HF HRV power *r* = 0.63, *p* = 0.0044 and for RMSSD *r* = 0.5, *p* = 0.013).

**Figure 6:**
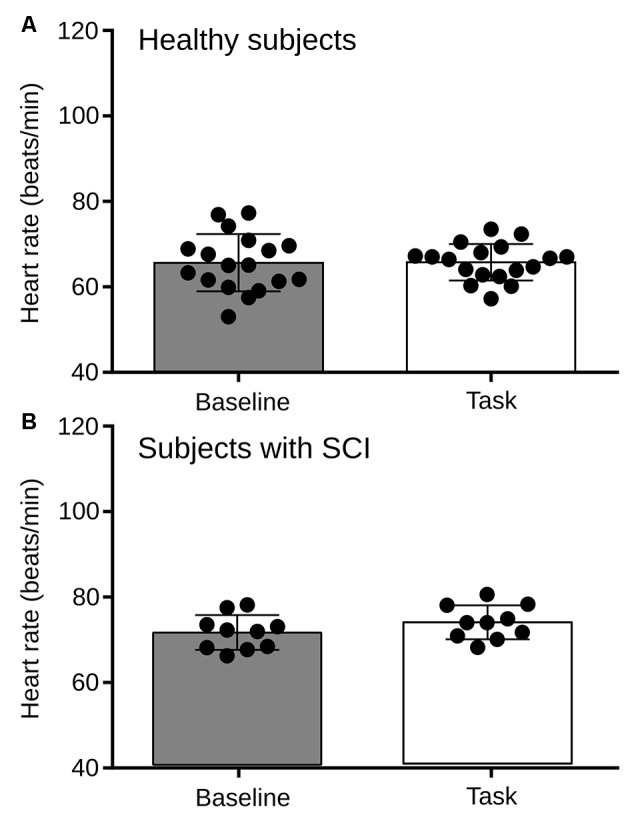
**(A)** Comparison of heart rate between the basal condition and the RMET task for Healthy subjects. No statistical differences were found in HR between the rest and the task (*p* = 0.9246). **(B)** Heart rate between the basal condition and the RMET task for subjects with SCI. No statistical differences were found (*p* = 0.2558).

Although the basal levels of LF/HF HRV and RMSSD were significantly lower in SCI subjects, we also explored the relation between answer certainty in the RMET and autonomic measures. Unlike healthy controls, the slopes of the correlations were negative, but not significant. Again, these results demonstrate, that in contrast to healthy individuals, SCI patients does not exhibit the normal autonomic changes expected during this social cognitive task.

## Discussion

This work presents evidence supporting that subjects with SCI have an alteration in the mechanism of autonomic regulation in front of a social cognition task. Anatomical injury level or residual motor and sensory function are not always indicative of the extent or pattern of autonomic failure (Bunten et al., 1998). In this context, it has long been described that autonomic disturbances in patients with SCI are mainly due to alterations of the sympathetic system, and that this sympathetic decentralization may cause a decreased capacity of the ANS rapid adaptation response to various environmental stimuli (Bunten et al., 1998; Claydon and Krassioukov, 2008). However, to date, there have been few reports that investigate alterations in parasympathetic activity in patients with SCI and its impact on physical and cognitive functions. Some studies have described that parasympathetic cardiac autonomic dysfunction in people with SCI below T6 can occur due to immobility, associated with impairment of the venous pump of the paralyzed muscles, which in turn, is associated with confinement in a wheelchair and a sedentary lifestyle (Serra-Año et al., 2015).

### Spinal Cord Injury, Autonomic Nervous System and Social Cognition

It is well established that individuals with SCI are prone to mood disorders and psychiatric diseases during chronic stages of their condition (Craig et al., 2015b). And it has also been widely reported that autonomic alterations may contribute to the prevalence of these disorders (Bal et al., 2010; Kemp et al., 2010). In our work, the group of subjects with SCI had no psychiatric disorders, which was part of the inclusion criteria for the study, and also this group of people was in a chronic stage of their condition (more than a year after the lesion).

Research in cardiovascular activity and autonomic regulation suggests that parasympathetic activity is instrumental for the efficient functioning of the ANS in complex environments and during social cognition tasks (Thayer and Lane, 2000; Park et al., 2012). Our results show that persons with SCI have a decrement in autonomic flexibility, measured by HRV, and also confirms information reported in previous studies and allows us to say that autonomic activity, measured by HRV, can be considered as a biomarker for the ability of humans to recognize emotions in others (Pumprla et al., 2002; Park et al., 2012).

HRV reflects parasympathetic and sympathovagal activity, which is increased in healthy subjects and is correlated with better certainly during RMET. This conjunction of facts may be explained by two theoretical models; the polyvagal theory and the Neurovisceral integration model. These models suggest that through the activity of the vagal system and the prefrontal cortex respectively exerts an inhibitory influence on the sinoatrial node reducing its activity and favoring a physiological resting state (increase HRV). This has been considered as favorable for interaction with the environment and social cognition functions (Thayer and Lane, 2000; Amodio and Frith, 2006).

### Behavioral Performance

Despite the fact that subjects with SCI had a similar performance in the task compared with the healthy subjects, there was a low level of certain responses in the group of persons with SCI compared to control group. It is important to note that while subjects performed the task they had no feedback associated with their performance, so they did not know if their answer was correct or not. The fact that the subject should report the level of certainty in the responses represented the subject’s subjective feeling about its performance and comfort level with the test, in this regard subject that expresses some degree of difficulty in performing this task, which assesses the affective component of the TOM, will respond with less certainty compared with a subject that feel comfortable during the test. We think that these low levels of certainty showed by subjects with SCI during RMET, reflects social cognition problems which may increase during stress situations. In this context it has been reported that the probability of having mood disorders is associated with high level of anxiety and low levels of self-efficacy, that is related to the confidence in their own ability to complete the task and reach goals (Munce et al., 2015).

Contrary to expectation, the ability of subjects to recognize the emotional state showed in the pictures during the test was similar in both groups (Figure 2A). Previous reports have shown that low levels of parasympathetic activity (measured by HRV) is correlated with poor performance in tasks that assess topics related to social cognition (Lee et al., 2005; Bal et al., 2010; Kemp et al., 2010; Quintana et al., 2012). However, it is important to note that most of the studies that have linked social cognition and HRV have been reported in subjects with psychiatric disorders, which could increase the behavioral difference between patients and controls.

### Differences in the Mechanism of Autonomic Regulation between Persons with SCI and Healthy Subjects

The fact that the group of subjects with SCI presents a significantly lower sympathovagal activity than the healthy population suggests that this group of people will have limitations in autonomic flexibility which may affect their ability to generate a functional autonomic balance in different situations (Rodrigues et al., 2016). For the other side, the group of healthy subjects changes their sympathovagal activity significantly during the execution of the RMET regarding the resting condition (Figure 4A), which it was not observed in the group of subjects with SCI (Figure 4B). These findings suggest that autonomic activity in healthy subjects compared to subjects with SCI is regulated differently during an emotion recognition task. These results are consistent with outcomes reported by studies that have promoted the use of drugs in psychotherapy that favors improvements in different topics of social cognition. These studies indicate that bounded dose of 3, 4-methylenedioxymethamphetamine (MDMA) optimize functions related to social cognition through a depressed activity of the amygdala (Bedi et al., 2009), increased levels of the hormone oxytocin (Dumont et al., 2009), decreased sympathetic activity and parasympathetic activity increased (Frye et al., 2014).

It has been estimated that around 55% of SCI subjects have difficulties in social participation (Craig et al., 2015b), is not clear which aspects are related to the ability of subjects to get over to this condition, but some aspects such as self-efficacy and low levels of negative moods contribute to resilience associated with increased level of well-being (Craig et al., 2015a; Guest et al., 2015). For the other side, anxiety, secondary complications, cognitive impairment, premorbid psychological disorder are related with both social stress and humor disorders, but other neurophysiological aspects such as autonomic flexibility have been poured described (Craig et al., 2015a). The different sympathovagal response but the similarity in the behavioral performance observed between control, and SCI subjects could be associated with low demands gathered by the test used. The lower certainty levels reported by SCI subjects during the social cognition task could be related to the difficulty to make autonomic regulation in response to environmental stimuli, which in turn can be caused by a malfunction of cortical networks linked with social cognition functions (Neurovisceral integration model). These results allow us to suggest that autonomic disturbances may affect important cognitive functions for adaptation and social reintegration, which corresponds to a fundamental stage in the rehabilitation process of patients with SCI.

We also consider some limitation of our study. First, although we obtained statistically significant results and the size effect was large, a large number of SCI subjects would have been desirable to compare with the larger number of controls. Also, because there is no control for the RMET task, the low level of certainty may be a general aspect of individuals with SCI, and the significance of this fact was not evaluated. Finally, broader aspects of social cognition were not included thus no contextualization of this particular feature in no possible here.

Different models have been proposed to explain the contribution of ANS to emotional processing and how HRV reflect higher cognitive functions as social cognition. The polyvagal theory and the Neurovisceral Integration model (Thayer and Lane, 2000) propose that the ANS, through prefrontal and vagal tone activity, favor an adequate interaction between a subject and their environment. This influence is achieved through an inhibitory effect on the sino-atrial node (Appelhans and Luecken, 2006) with a critical role for parasympathetically mediated inhibition of autonomic arousal in emotional expression. This modulation could be observed throughout the HRV measures, which is informative about individuals capacity for regulated emotional responding and functions related to social cognition (Lane et al., 2009; Thayer and Lane, 2009; Quintana et al., 2012).

The neurovisceral integration model focuses on the inhibitory role of the prefrontal cortex and their contribution to modulate cardiovascular function through parasympathetic vagal pathways (Thayer and Lane, 2000). This model establishes the idea that some topics of cognitive and emotional function could be observed throughout HRV measures (Thayer and Lane, 2000). To explain this fact, the neurovisceral integration model describes that the prefrontal, cingulate and insula cortices form an interconnected network with bi-directional communication with the amygdala (Thayer and Lane, 2000; Quintana et al., 2012). The amygdala is under tonic inhibitory control via prefrontal vagal pathways to intercalated cells. Impairment in the prefrontal cortex—for example in depression or autism—leads to disinhibition of the central nucleus of the amygdala and medullary cardio acceleratory circuits, thus leading to increased heart rhythm and decreased HRV. In this way, HRV may be related to social cognition given its relationship with activity in prefrontal neural structures (Lane et al., 2009; Thayer and Lane, 2009; Quintana et al., 2012).

### Coda

In summary, this research provides direct evidence to support the relationship between ANS’ function and emotion recognition, a facet of social cognition, in subjects with SCI. Our results show that patients with SCI have problems in autonomic flexibility that may influence negatively on topics related to social cognition during its social and labor reinsertion process.

## Author Contributions

GV-D and PEM designed the study. GV-D and GR-L carried out the study and collected the data. EPB and GV-D analyzed the data. GV-D, EPB, GR-L and PEM wrote the manuscript.

## Conflict of Interest Statement

The authors declare that the research was conducted in the absence of any commercial or financial relationships that could be construed as a potential conflict of interest.
